# The nonlinear association between apolipoprotein B to apolipoprotein A1 ratio and type 2 diabetes: Erratum

**DOI:** 10.1097/MD.0000000000006541

**Published:** 2017-03-24

In the article “The nonlinear association between apolipoprotein B to apolipoprotein A1 ratio and type 2 diabetes”,^[1]^ which appeared in Volume 96, Issue 1 of *Medicine*, the authors’ affiliations were listed incorrectly. The correct affiliations are as follows:

Yong Mao, Department of Epidemiology and Health Statistics, School of Public Health, Kunming Medical University, Kunming, China

Yang Xu, Department of Anesthesiology, Xiangyang No. 1 People's Hospital, Hubei University of Medicine, Xiangyang, China

Leihong Lu, Department of Dermatology, Linyi People's Hospital, Linyi, China.
